# A Comprehensive Review of Techniques for Enhancing Zirconia Bond Strength: Current Approaches and Emerging Innovations

**DOI:** 10.7759/cureus.70893

**Published:** 2024-10-05

**Authors:** Abdulrahman S Al-Amari, Mashael S Saleh, Abdullah A Albadah, Abeer A Almousa, Waleed K Mahjoub, Rasha M Al-Otaibi, Essa M Alanazi, Atheer K Alshammari, Abdulrahman T Malki, Khalid F Alghelaiqah, Lamya F Akbar

**Affiliations:** 1 Family Dentistry, Ministry of Health, Riyadh, SAU; 2 Dentistry, Vision College, Jeddah, SAU; 3 Dentistry, Umm Al-Qura University, Makkah, SAU; 4 General Dentistry, One Med Medical Polyclinic, Riyadh, SAU; 5 Dentistry, King Saud University, Riyadh, SAU; 6 Dentistry, University of Hail College of Dentistry, Hail, SAU; 7 Dentistry, King Saud bin Abdulaziz University, Riyadh, SAU; 8 Dentistry, Ministry of Health, Al Bahah, SAU

**Keywords:** 10-mdp primers, polymer-infiltrated ceramic networks (picn), resin cements, surface treatment, zirconia bonding

## Abstract

The increasing use of zirconia in dental restorations necessitates a comprehensive understanding of effective bonding techniques to ensure long-term clinical success. Zirconia's unique chemical composition presents challenges in achieving a durable bond as it lacks the glass phase necessary for traditional etching and silanization processes. This review evaluates current methods and emerging innovations for enhancing zirconia bond strength to resin cements. Our findings emphasize the importance of mechanical surface treatments such as air-particle abrasion and tribochemical silica-coating, which significantly improve micromechanical retention. Laser irradiation, while less commonly used, also shows promise in enhancing bond strength without compromising zirconia's structural integrity; 10-methacryloyloxydecyl dihydrogen phosphate (10-MDP) primers emerged as critical agents in forming stable P-O-Zr bonds, particularly when used with resin cements containing 10-MDP. However, variations in formulation and application methods impact their overall efficacy. Resin cement demonstrated superior bond strength compared to conventional cement, but clinical outcomes varied, highlighting the importance of cement-primer compatibility and strict procedural adherence. Emerging technologies such as polymer-infiltrated ceramic networks (PICNs) and additive manufacturing (AM) of zirconia offer potential for future advancements, although they require further research to address mechanical and aesthetic challenges. In conclusion, while established methods such as sandblasting and the use of MDP primers remain reliable, ongoing research into novel materials and techniques continues to offer opportunities for enhancing zirconia bonding. Clinicians must balance effectiveness, procedural complexity, and clinical practicality when selecting the most appropriate bonding protocols for zirconia restorations.

## Introduction and background

Gracis et al. proposed a classification for restorative ceramics in 2015, which has since become widely adopted in dental literature [1]. Zirconia, or zirconium oxide, was classified as a polycrystalline ceramic material with three different crystal structures: monoclinic, tetragonal, and cubic, depending on temperature and pressure conditions. Each form possesses distinct mechanical and optical properties, making zirconia suitable for various clinical applications [2]. The initial introduction of zirconia into dentistry primarily utilized the tetragonal phase, stabilized at room temperature by adding 3 mol% yttrium oxide (3Y-TZP, yttria-stabilized tetragonal zirconia polycrystal). This early 3Y-TZP was valued for its dense microstructure and high flexural strength, although it exhibited low esthetic translucency due to its opacity [3].

As material science advanced, modifications were made to the zirconia composition to improve its esthetic characteristics. Researchers increased the percentage of yttrium oxide, which enhanced the stabilization of the cubic phase of zirconia, a non-birefringent phase that offers improved translucency compared to traditional 3Y-TZP, making it more suitable for esthetically demanding cases [3].

Adhesive dentistry has revolutionized clinical outcomes by enabling better retention of fixed prostheses, even in situations where abutments have limited height, taper, or surface area [4]. A critical factor in this success is the creation of a strong bond between three interfaces: the ceramic restoration, the adhesive dental cement, and the tooth structure. This robust bond forms what is commonly referred to as a "monobloc" effect, ensuring not only the esthetics of the restoration but also its long-term functionality and durability [5].

However, despite the evolution of zirconia materials to levels that now rival the esthetics of glass ceramics, achieving consistent and durable bonding of zirconia restorations to tooth structure remains a significant challenge for clinicians. Numerous studies have investigated methods for enhancing zirconia bond strength, yet no definitive gold-standard technique has emerged from the literature. This comprehensive review aims to address this gap by presenting an in-depth examination of the current techniques, materials, and emerging innovations used to improve the bond strength between zirconia restorations and tooth structures. By exploring both well-established and novel bonding strategies, this review seeks to provide clinicians with a clearer understanding of how to optimize zirconia adhesion in their restorative practice.

## Review

The bonding procedure to zirconia

From a materials science perspective, polycrystalline ceramics are inorganic, non-metallic materials that do not have a glass phase [1]. The lack of silica in the microstructure makes etching by strong acids and alkalis difficult. The roughening of the surface, which is essential for mechanical retention, is therefore compromised. Surface hydroxyls, which are formed by silane coupling agents for chemical bonding, are also not possible due to the absence of silica [6]. In a review article, Chatterjee and Ghosh reported that zirconia surface pretreatment is essential to increase its bond strength [7], and they suggested mechanical, chemical, and mechanochemical methods of pretreatment. Furthermore, mechanochemical pretreatment techniques are recommended for zirconia restorations. Micromechanical retention serves as an adjunct to chemical bonding, especially because of the materials' affinity for oral fluids such as saliva, blood, and salivary proteins [4]. It is likely that chemical bonding alone may lead to the debonding of restorations in the oral environment.

In cases where the abutment teeth have adequate retention and resistance form, conventional luting cements can be used. However, bonding of the restorations is required in patients with compromised retention forms. Since concerns about the debonding of zirconia have increased over the past few years, the APC (A=airborne-particle abrasion (APA), P=primer, and C=resin cement) protocol suggested by Blatz et al. has been recommended for the clinical success and longevity of zirconia restorations [6]. Al-Akhali et al. suggested that this protocol be performed by clinicians chairside after decontaminating the intaglio surface of the prosthesis [8]. Research has indicated that APA, followed by vigorous agitation of the zirconia primer, creates a positively charged surface with surface energy suitable for chemical bonding with resin cement. The following sections explain in detail the three parameters of the APC concept. To render zirconia more "bondable," researchers have attempted to infiltrate polymers or silica in the porous structure of zirconia over the last few years. These hybrid ceramics are yet to be used in clinical settings, as results of long-term clinical studies are awaited. Once these materials become feasible to use clinically, combining the best restorative dentistry materials, i.e., ceramics and composites, would truly give our patients optimal indirect restorations.

Surface decontamination and mechanical treatments

Saliva contains organic materials, such as proteins and enzymes, that reduce surface energy and impair mechanical retention essential for bonding. Therefore, surface decontamination is crucial for achieving strong and durable bonds between zirconia restorations and resin cements, especially after intraoral contamination from saliva [9]. Various surface decontamination methods have been studied, with mechanical surface treatments consistently showing superior results. In clinical practice, phosphoric acid (H_3_PO_4_) is commonly used due to its ability to dissolve organic contaminants. However, its effectiveness decreases significantly after thermocycling, which is a process that simulates material aging [9,10]. Moreover, as phosphoric acid often leaves phosphorus residues that interfere with bonding, it is less suitable for zirconia surfaces, particularly when long-term bond stability is critical [10].

In contrast, Ivoclean, a zirconium oxide-based cleaner, mechanically removes organic contaminants and maintains bond strength even after aging [9]. Although scanning electron microscopy (SEM) analysis shows that Ivoclean may leave behind zirconium oxide particles, these particles do not negatively affect bond strength and may even enhance micromechanical retention [9-13]. Ivoclean, therefore, acts both as a decontaminant and as a promoter of mechanical retention, making it a reliable choice for ensuring long-term bond success after thermocycling. Other cleaning agents, such as isopropanol and water rinsing, show limited efficacy in fully removing contaminants and restoring bond strength [10]. Instead, advanced solutions such as Katana Cleaner and ZirClean more effectively remove organic contaminants and restore bonding strength [10]. However, these methods are still secondary to mechanical treatments when it comes to achieving long-term bond durability.

Among various mechanical treatments, APA has consistently demonstrated superior performance in achieving high bond strength comparable to that of uncontaminated surfaces. APA works by increasing surface roughness, promoting micromechanical retention, and improving adhesion [10-13]. However, APA also has its risks; excessive air pressure can introduce microcracks and potentially compromise zirconia's long-term mechanical durability, especially in load-bearing restorations [10,13]. In comparison, sodium hypochlorite, while useful as a disinfectant, does not restore bond strength as effectively as zirconia-specific cleaners such as Ivoclean. This highlights the importance of using specialized decontaminants designed for zirconia rather than general-purpose disinfectants [10]. Alkaline treatments such as sodium hydroxide (NaOH) and zirconium hydroxide (Zr(OH)_4_) have also shown potential. Faeze et al. [11] found that Zr(OH)_4_ significantly improved bonding by increasing surface energy and chemical reactivity, especially when applied at elevated temperatures. Similarly, Qian et al. [12] demonstrated that nanoscale alkaline coatings, such as NaOH and nano-Zr(OH)_4_, enhanced the shear bond strength (SBS) of zirconia and provided a stable bond under moist conditions. Flores-Ferreyra et al. [13] reported that APA combined with NaOH produced the highest bond strength, while APA with hydrofluoric acid (HF) significantly reduced bond strength, indicating that chemical and mechanical treatments must be carefully chosen to avoid negative interactions. Lorenzoni et al. [14] also supported the use of NaOH combined with 10-MDP primers and found that it significantly improved bond strength by increasing surface energy and promoting chemical bonding with resin cement.

A systematic review and meta-analysis by Thammajaruk et al. [15] evaluated various decontamination methods and found that mechanical methods, particularly alumina air-abrasion, outperformed chemical methods in restoring bond strength and enhancing micromechanical retention [15]. Alumina air-abrasion roughened the surface to produce bond strengths of 25.18 MPa, while plasma treatment demonstrated even higher bond strengths of 30.81 MPa. Both methods significantly improved mechanical retention by modifying the surface structure, which is essential for achieving strong and durable bonds [15]. For long-term success, mechanical surface treatments such as air abrasion remain critical. Sodium hypochlorite is useful for short-term decontamination but leads to lower bond strengths over time, while air abrasion consistently maintains bond strength under aging conditions [15,16]. Feiz et al. [17] further highlighted that APA is one of the most reliable methods for zirconia decontamination, as it not only removes contaminants but also enhances both mechanical and chemical bonding by increasing surface roughness [17]. In contrast, phosphoric acid etching left phosphate residues that interfered with the bonding of 10-MDP, a critical component in chemical adhesion to zirconia [17]. This makes phosphoric acid unsuitable for contaminated zirconia surfaces. Although Ivoclean is effective in the short term, it leads to reduced bond strength after aging, reinforcing the need for mechanical treatments to ensure long-term bond success [17].

Koko et al. [18] investigated the bond strength of resin cement on saliva-contaminated zirconia. The study found that APA followed by primer application preserved bond strength even after thermocycling, underscoring the importance of mechanical decontamination for ensuring durable chemical bonding [18]. Similarly, Demir et al. [19] demonstrated that alumina blasting, particularly when paired with 10-MDP-containing resin cements, was the most effective mechanical treatment for restoring bond strength. Traditional methods, such as air-water spray and pumice cleaning, were far less effective, confirming the superiority of mechanical methods [19]. Air abrasion using aluminum oxide particles plays a pivotal role in enhancing surface roughness, promoting micromechanical retention, and improving adhesion. When combined with 10-MDP primers, this treatment optimizes bond strength by improving both mechanical retention and chemical bonding [20]. This is further supported by findings from Hajjaj et al. [21], whose comparison of eight cleaning protocols demonstrated that APA restored SBS to levels comparable to uncontaminated zirconia, reinforcing its role as a reliable method for enhancing bonding outcomes [21]. Table 1 shows surface decontamination methods for enhancing zirconia bonding.

**Table 1:** Surface decontamination methods for enhancing zirconia bonding SEM: scanning electron microscopy; HF: hydrofluoric acid; APA: airborne-particle abrasion; MDP: methacryloyloxydecyl dihydrogen phosphate

Study	Decontamination Method	Mechanism	Effectiveness in Restoring Bond Strength	Remarks
Borges et al. [9]; Sulaiman et al. [10]	Phosphoric acid	Dissolves organic contaminants	Less effective after thermocycling; leaves phosphorus residues	Leaves phosphorus residues, reducing adhesive penetration and bond strength.
Borges et al. [9]; Flores-Ferreyra et al. [13]	Ivoclean (zirconium oxide-based)	Mechanically removes organic contaminants, leaves zirconium oxide particles	Effective in maintaining bond strength even after aging	SEM shows particles may enhance micromechanical retention.
Sulaiman et al. [10]	Isopropanol and water rinsing	Removes superficial contaminants	Limited efficacy in fully removing contaminants	Insufficient for deeper decontamination.
Sulaiman et al. [10]	Katana cleaner and ZirClean	Advanced cleaning agents targeting organic contaminants	Effective in removing organic contaminants and restoring bond strength	Addresses deeper contaminant penetration.
Flores-Ferreyra et al. [13]; Sulaiman et al. [10]	Airborne-particle abrasion (APA)	Creates surface roughness through mechanical action	Consistently high bond strength but risk of surface flaws like microcracks	May introduce microcracks affecting long-term durability.
Sulaiman et al. [10]	Sodium hypochlorite	Combined mechanical and chemical treatment	Restores bond strength close to uncontaminated levels	Less effective than zirconia-specific cleaners.
Zavare et al. [11]; Lorenzoni et al. [14]	Sodium hydroxide and zirconium hydroxide	Increases surface energy and chemical reactivity	Improves bonding by increasing surface energy and chemical reactivity	Effective when combined with heat-assisted decontamination.
Qian et al. [12]	Sodium hydroxide and nano-zirconium hydroxide coatings	Nanoscale particle size allows for deeper surface penetration	Enhanced shear bond strength, stable under moist conditions	Durable bond even in challenging clinical conditions.
Flores-Ferreyra et al. [13]	APA with sodium hydroxide (NaOH)	Combined mechanical and chemical treatment	Highest bond strength achieved	Effective, but APA with HF reduces bond strength.
Flores-Ferreyra et al. [13]	APA with hydrofluoric acid (HF)	Combined mechanical and chemical treatment	Reduced bond strength	Detrimental combination for long-term adhesion.
Lorenzoni et al. [14]	MDP-based primers (combined with APA)	Promotes chemical bonding via phosphate groups and increases surface energy	Significantly improved bond strength	Best performance when combined with mechanical treatments.

Chemical surface modifications: silica coating, multi-acid etching, and phosphate-based bonding (10-MDP)

Silica Coating and Silane Primers

A systematic review and meta-analysis by Kumar et al. [22] evaluated various surface treatment methods to enhance the SBS between zirconia and resin cement, focusing primarily on silica coating and air abrasion (sandblasting). Silica coating, which involves blasting zirconia with silica-coated alumina particles to create micro-roughness and deposit a silica layer, was found to be the most effective treatment. This silica layer chemically interacts with silane coupling agents, producing strong chemical bonds and enhanced micromechanical retention, leading to the highest SBS values before and after thermocycling [22].

Air abrasion also significantly increased SBS by roughening the zirconia surface and enhancing micromechanical retention. However, unlike silica coating, air abrasion does not chemically modify the zirconia surface. While effective initially, air abrasion raises concerns about long-term stability due to its potential to induce a tetragonal-to-monoclinic phase transformation, which can weaken zirconia's mechanical properties over time. This transformation, combined with the risk of microcracks, particularly in load-bearing restorations, makes air abrasion less durable compared to silica coating [22,23]. Souza et al. [24] highlighted that while APA is effective at increasing surface roughness, it may negatively impact the long-term mechanical properties of zirconia due to phase transformation, emphasizing the need to manage surface roughness carefully [24]. Due to its combined mechanical and chemical bonding capabilities, silica coating consistently outperformed air abrasion in both initial adhesion and long-term durability. Tribochemical approaches, such as silica coating, which blends mechanical roughening with chemical interaction, have been shown to provide more reliable, long-lasting bonds [22]. Supporting this, Iwasaki et al. [23] demonstrated that silica-coated alumina particles provided significantly higher bond strength compared to traditional air abrasion, particularly after thermocycling [23]. Tribochemical silica coatings have continued to outperform mechanical-only methods such as air abrasion. Özcan's [25] study showed that silica-coated particles achieve both micromechanical retention and chemical bonding, minimizing subsurface cracks and enhancing adhesion through silane coupling agents [25]. Nagaoka et al. [26] also highlighted the importance of achieving complete silica layer coverage to ensure consistent bonding outcomes, noting that gaps in the silica layer could reduce bond strength [26]. Direct comparisons between air abrasion and tribochemical silica coating consistently show that the combination of mechanical and chemical bonding in silica coating produces stronger and more durable bonds in zirconia restorations [26].

Araújo et al. [27] found that silicatization followed by silanization significantly improved bond strength due to enhanced surface energy and wettability. In contrast, multi-mode adhesives, although providing adequate bond strength, were less stable than silica-silane combinations, demonstrating the superiority of tribochemical treatments in clinical settings that demand long-term durability [27]. The timing of surface treatments is also crucial; Ebeid et al. [28] observed that air abrasion was more effective for post-sintered zirconia, especially after aging, while silica coating was less effective when applied in the pre-sintered stage [28]. Overall, while air abrasion remains a dependable method for increasing bond strength, the incorporation of chemical bonding through silica coating or selective infiltration etching (SIE) offers superior, longer-lasting results, particularly under aged conditions [29].

Tribochemical silica coating and SIE significantly enhance zirconia-resin bonding by combining mechanical and chemical retention, offering superior durability over mechanical treatments alone [30,31]. Ultrashort-pulse laser (UPL) treatments also enhance bond strength by precise surface roughening without damaging zirconia [32]. This dual action of mechanical roughening and chemical interaction provides a robust solution for enhancing zirconia-resin bonding, making these methods highly effective for clinical applications.

The Zircos-E etching system: a multi-acid approach to zirconia surface treatment

The Zircos-E system was developed to address the bonding challenges associated with zirconia ceramics, which lack the silica phase essential for traditional bonding methods. Conventional treatments such as hydrofluoric acid and silane coupling agents often fall short on zirconia. In contrast, the Zircos-E system utilizes a multi-acid composition to create microporous surfaces that enhance micromechanical interlocking between zirconia and resin cements, significantly improving bond strength [33,34]. The etching process involves immersing zirconia specimens in the Zircos-E solution for approximately two hours, followed by thorough rinsing. This treatment ionizes the zirconia surface, creating a roughened and more reactive topography that promotes higher SBS compared to untreated zirconia or zirconia treated solely with air abrasion [33]. Key observations from SEM and atomic force microscopy (AFM) revealed that the Zircos-E system produces uniform surface roughness with small micropores, which increase micromechanical retention without causing significant material loss or damage, a common issue with more aggressive treatments such as air abrasion. The controlled roughness maintains the structural integrity of zirconia while enhancing adhesion, unlike mechanical treatments that can cause microcracks or surface damage. When combined with air abrasion, particularly on translucent zirconia, the Zircos-E etching system demonstrated even higher bond strength values. This dual-action approach of chemical etching and mechanical roughening maximizes bond strength and overcomes the limitations of either method when used independently, enhancing the longevity and performance of zirconia-based restorations [33].

Testing on monolithic zirconia samples followed by resin cement application showed that Zircos-E etched surfaces promoted stronger adhesion compared to untreated. The use of specific primers, such as MKZ Primer and Monobond Plus, further enhanced the adhesive bond, contributing to improved bond durability [34]. This combination of Zircos-E with advanced primers underscores the importance of multi-step surface treatments in achieving optimal bonding outcomes. Zircos-E etched samples, particularly those treated with primers, maintained significantly higher bond strength than non-etched or sandblasted groups, indicating improved long-term stability of the bond and resistance to degradation caused by temperature variations and moisture exposure [34].

In a study by Cho et al. [35], the Zircos-E etching system was further evaluated and compared to other common surface treatments, such as air abrasion and tribochemical silica coating. The study found that a two-hour application resulted in the highest surface roughness and improved resin tag infiltration, significantly enhancing SBS between zirconia and resin cements. Super-Bond C&B-treated samples exhibited the highest SBS values at 16.15 MPa, while Panavia F 2.0 showed lower values after Zircos-E treatment. The ability to optimize bond strength through controlled etching times further demonstrates the flexibility of the Zircos-E system and its suitability for clinical applications. Failure mode analysis revealed that Zircos-E-treated samples exhibited a mix of cohesive and adhesive failures, indicating a strong adhesive interface, whereas other methods showed more adhesive failures, suggesting weaker bonds at the zirconia-resin interface [35].

Overall, the Zircos-E etching system effectively enhances the bond strength of resin cements to zirconia by creating a retentive and chemically active surface. Its multi-acid solution improves both surface roughness and bond durability, making it a promising tool for clinical applications in zirconia restorations. Zircos-E outperforms traditional methods such as air abrasion, maintaining bond strength over time, even under thermocycling conditions, making it a reliable option for improving zirconia bonding. Further evaluation is recommended to assess its long-term clinical success, particularly in various zirconia-based restorations where bonding challenges persist [33-35].

Phosphate-based bonding: the role of 10-MDP in zirconia adhesion

Combining 10-MDP primers with mechanical surface treatments such as APA significantly enhances zirconia-resin bonding. Studies have shown that specimens treated with abrasion followed by the application of 10-MDP-containing primers, such as Z-PRIME Plus, exhibited the highest SBS values, attributed to pronounced micromechanical interlocking and effective resin infiltration [36]. Shin et al. [37] confirmed that this combination resulted in higher SBS compared to untreated groups and those treated with tribochemical silica coating, highlighting the unique advantage of phosphate-based bonding over silica-silane systems for zirconia [37]. The chemical structure of 10-MDP significantly enhances adhesion between zirconia and resin-based cements. Sandblasting followed by 10-MDP application improved bonding performance, with cohesive and mixed failure modes observed in treated groups, indicating its efficacy in promoting durable adhesion [38].

Durability and Hydrolytic Resistance of 10-MDP Bonds

A key advantage of 10-MDP is its resistance to hydrolytic degradation. Primers such as Clearfil Ceramic Primer and Monobond Plus, both containing 10-MDP, consistently provided superior bond strength even after thermocycling and long-term water storage. This durability is crucial in clinical settings where maintaining bond strength over time is essential [39]. A study on the effects of solvents, such as acetone, found that they further enhance 10-MDP's chemical affinity with zirconia, optimizing bonding outcomes [40].

Advanced Analysis of 10-MDP Interaction

Advanced techniques such as the time-of-flight secondary ion mass spectrometry (ToF-SIMS) have confirmed the formation of stable P-O-Zr bonds on 10-MDP-treated zirconia surfaces. These strong bonds contribute to the material's durability, enhancing resin cement flow and adhesion even after mechanical aging [41,42]. However, studies indicate that using MDP in conjunction with silane may reduce bond strength due to competitive interactions, underscoring the importance of selecting appropriate primers [43].

Optimizing 10-MDP Primer Applications

Research indicates that applying 10-MDP primers twice achieves the highest SBS values, while further applications do not enhance and may even reduce bond strength, highlighting the need for precise application techniques in clinical settings [44]. The effectiveness of 10-MDP extends beyond zirconia to other core materials such as amalgam and composite resin, although its performance is less effective on metallic cores such as nickel-chromium (Ni-Cr) [45]. Systems such as Clearfil Ceramic Primer with Clearfil SA Cement demonstrate superior bonding with Ni-Cr cores, emphasizing the importance of selecting suitable primer-cement combinations for reliable outcomes [46]. Overall, 10-MDP-containing primers consistently deliver superior bonding strength and durability, particularly when combined with mechanical surface treatments. The strong chemical interaction between 10-MDP and zirconia's oxide layer, along with enhanced mechanical retention, ensures durable bonds that resist hydrolytic degradation, making them an essential component in clinical applications [38-46].

*Comparison Between 10-MDP and Other Chemical Modifications* The distinction between 10-MDP-based treatments and other chemical surface modifications, such as silica coating and Zircos-E multi-acid etching, lies in their specific bonding mechanisms. In the case of silica coating, the silica particles chemically interact with silane coupling agents. The silica layer enhances micromechanical retention by roughening the zirconia surface, while the silane chemically bonds to the resin cement. This dual mechanism primarily strengthens chemical bonding through silane, which helps improve the adhesion between the zirconia surface and the resin cement [22]. On the other hand, Zircos-E multi-acid etching works by roughening the surface using a blend of acids. This process increases micromechanical retention by creating a reactive and microporous surface, allowing better resin infiltration. However, the acids in this system do not directly engage in a chemical bonding interaction with the resin cement, as seen with 10-MDP or silane systems. Instead, their primary role is to provide mechanical retention through surface roughening [33-35].

The 10-MDP phosphate monomer operates differently by chemically interacting with zirconia through its phosphate group, which bonds to the oxide layer on the zirconia surface. This interaction forms a stable P-O-Zr bond, which is further complemented by the methacrylate group bonding to the resin cement. The unique advantage of 10-MDP lies in its ability to form strong, stable bonds without relying on silica or multi-acid etching systems. Compared to other bonding agents, 10-MDP has demonstrated superior resistance to hydrolytic degradation, making it highly effective for long-term clinical applications [36-46].

In summary, 10-MDP provides a unique phosphate-based chemical bonding mechanism that works well in conjunction with mechanical roughening techniques such as APA. Unlike silica coatings, which primarily depend on silane for chemical bonding, or multi-acid etching, which focuses on surface roughening for retention, 10-MDP directly targets zirconia's oxide layer to form a durable chemical bond. This makes 10-MDP a superior choice to overcome the hydrolytic and mechanical challenges associated with zirconia bonding. Table 2 compares chemical treatments for zirconia bonding.

**Table 2:** Overview of chemical surface treatments for enhancing zirconia-resin bonding MDP: 10-methacryloyloxydecyl dihydrogen phosphate; P-O-Zr: phosphate-oxygen-zirconium bonds

Study	Chemical Surface Treatment	Mechanism	Effectiveness	Advantages	Disadvantages
Kumar et al. [22]	Silica coating (TSC)	Blasting with silica-coated alumina particles, introducing a silica layer that bonds chemically with silane agents.	High bond strength, especially with silane agents, effective before and after aging.	Combines mechanical and chemical retention for strong, durable bonds.	Requires silane primers for full effectiveness, can be costly.
Özcan [25] Nagaoka et al. [26]	Tribochemical silica coating (TSC)	Deposits a silica layer on zirconia and uses silane coupling agents for chemical bonding.	Outperforms air abrasion; improves bond strength with MDP primers.	Enhanced chemical bonding and micromechanical retention; better long-term durability.	Incomplete silica deposition can affect bond strength.
Rigos et al. [30]	Selective infiltration etching (SIE)	Glass-containing agents infiltrate zirconia grain boundaries, creating a highly retentive surface for strong mechanical interlocking.	It ranks highest for both immediate and long-term bond strength and outperforms other methods post-aging.	Highest long-term bond durability, strong mechanical interlocking.	Complex processes may require specialized equipment.
Rigos et al. [30]	Hot etching	It creates nanoscale roughness using an acidic solution, improving surface roughness, but it involves safety concerns due to corrosive chemicals.	It is effective in creating roughness, but concerns over safety and consistency limit its application.	Effective roughening method creates nanoscale surface texture.	It involves the use of corrosive chemicals and requires caution.
Li et al. [29]	Gas plasma	Modifies surface through high-energy ion bombardment, increases polar group content but does not improve bond strength significantly.	Minimal improvement in bond strength, not recommended as a standalone treatment.	No significant structural damage increases surface energy.	Limited effectiveness, does not significantly enhance bonding.
Yilmaz-Savas et al. [52]	MDP-containing primers	Interacts with zirconia's oxide layer, enhancing both mechanical and chemical adhesion, forming stable P-O-Zr bonds.	Significantly improves bond strength, especially when combined with mechanical treatments.	Enhances both chemical and mechanical bonding, very durable.	Requires combination with mechanical treatments for best results.

Laser surface treatments: a comparative analysis

Laser surface treatments present a promising method of enhancing the bond strength between zirconia and resin-based materials. These treatments focus on increasing surface roughness and energy to improve both micromechanical and chemical adhesion. Among the lasers studied, the CO_2_ laser demonstrated superior results in terms of SBS. Operating through thermomechanical ablation, the CO_2_ laser creates a roughened surface with micro-retentive features, which is ideal for bonding [47]. The uniform roughness produced by the CO_2_ laser significantly enhances micromechanical retention and optimizes resin cement adhesion. Its precision, coupled with low-energy settings to avoid thermal damage, makes CO_2_ laser treatments highly effective in clinical applications where it is paramount to maintain zirconia's structural integrity.

The Nd laser, which operates by inducing micro-explosions on the zirconia surface, also increased surface roughness through localized melting of the outer layer. However, high-energy Nd laser settings were found to introduce microcracks, which could compromise long-term mechanical stability [47]. Although the Nd laser improved surface roughness and SBS at moderate energy levels, the risk of surface defects at higher settings underscores the importance of carefully controlling energy output to avoid compromising bond durability [47].

Er lasers were less effective than CO_2_ and Nd lasers in improving bond strength. While Er lasers created surface roughness through micro-explosions, the consistency and depth of roughness were insufficient to generate meaningful retention points. As a result, Er lasers failed to achieve statistically significant improvements in SBS compared to untreated zirconia, making them less suitable for applications where long-term bond durability is critical [47,48].

A comparison was done by Du and Niu [48] on Er and Nd laser treatments with traditional methods such as sandblasting and silica-zirconia (Si-Zr) coating. They found that both laser treatments increased surface roughness and micromechanical retention. However, the Er laser created larger pits and pores, which improved micromechanical interlocking. Nonetheless, the presence of microcracks in both Er and Nd laser-treated surfaces reduced long-term bond durability, limiting their clinical use [48]. In contrast, Si-Zr coating increased surface roughness and introduced silica, which chemically bonded with silane coupling agents. This coating yielded the highest bond strength among the tested groups. It highlights the advantages of combining mechanical roughening with chemical adhesion mechanisms, underscoring the limitations of purely mechanical laser treatments [48].

A study by Arami et al. [49] evaluated the effects of different laser types on zirconia ceramics. They found that Er lasers increased surface roughness effectively, especially at higher power outputs, with controlled surface irregularities and ablation depth. In addition, Nd lasers generated higher surface roughness than both Er and CO_2_ lasers, but their high-energy settings also produced microcracks, rendering them less ideal for clinical use. While CO_2_ lasers did create rough surfaces, they generated fewer cracks compared to Nd lasers. The findings highlight the importance of selecting appropriate power settings to balance surface roughness with avoiding structural damage [49].

Jauregui-Ulloa and Marocho [50] highlighted the effectiveness of femtosecond lasers in modifying zirconia surfaces for improved bonding. Femtosecond lasers operate with ultrashort pulse durations that minimize heat generation, allowing precise surface modifications without inducing phase transformations or causing structural damage. This precision makes femtosecond lasers highly effective for enhancing surface roughness and bond strength, offering a cutting-edge solution for restorative dentistry where maintaining the structural integrity of zirconia is crucial [50].

Sahoo et al. [51] explored the application of ultrashort pulse lasers (USPL), including femtosecond and picosecond lasers, for zirconia surface treatment. These lasers create precise surface textures without introducing defects such as microcracks, which are common with traditional methods. USPL's cold ablation process prevents thermal damage and can create highly controlled surface patterns that enhance both mechanical and chemical retention. This precision surpasses grit-blasting in terms of bond strength, making USPL a superior alternative for long-term zirconia-resin bonding [51]. Femtosecond lasers are particularly effective, as they create surface roughness without causing phase transformations or cracks, further enhancing their efficacy for surface modifications. However, their high cost and complexity may limit their use in clinical situations despite their precision and lack of thermal damage [47].

In a study by Yilmaz-Savas et al. [52], femtosecond laser technology was found to create precise microstructuring on zirconia surfaces without causing phase transformation or thermal damage. Femtosecond laser-treated surfaces exhibited well-defined surface pits that significantly enhanced micromechanical retention and bond strength [52]. The homogeneous distribution of resin cement on laser-treated surfaces further improved adhesive bonding, making femtosecond lasers a promising option for zirconia surface modification in clinical applications.

The combined use of femtosecond lasers and air abrasion, particularly when applied before and after sintering, produced significant improvements in both surface roughness and bond strength. Furthermore, femtosecond laser treatment before sintering resulted in the highest bond strength. This suggests that combining laser treatments with mechanical methods enhances the effectiveness of zirconia surface modifications, offering significant clinical potential for improving resin-zirconia bonding [53].

Finally, a comparison of femtosecond lasers with tribochemical silica coating (TBC) and other laser techniques found that the femtosecond laser created regular surface patterns that significantly improved bond strength without causing thermal damage or phase transformation. This precision distinguishes femtosecond lasers from other methods, such as Nd and CO_2_ lasers, which may introduce surface defects or thermal changes that could compromise the structural integrity of zirconia [54]. While CO_2_ lasers strike a balance between roughening the surface and avoiding structural damage, Nd lasers require careful energy management to minimize the risks of defects. By combining laser treatments with mechanical methods such as air abrasion or Si-Zr coating, clinicians can leverage both micromechanical retention and chemical bonding to achieve the best outcomes.

Laser treatments, such as CO_2_, Nd, Er, and femtosecond lasers, enhance zirconia-resin bonding by increasing surface roughness and energy. The CO_2_ laser excels in creating uniform roughness without thermal damage, making it highly effective for clinical use. Even though Nd lasers improve roughness, they can cause microcracks and phase transformations that potentially affect long-term stability. Er lasers, while increasing roughness, are less effective than CO_2_ lasers and may introduce surface defects. Femtosecond lasers offer precise surface modifications with minimal thermal damage and no phase transformation, providing superior bond strength and potential for clinical applications. Combining laser treatments with mechanical methods, such as air abrasion, further enhances bonding outcomes [31,47-54]. Table 3 shows the impact of various laser surface treatments on zirconia bonding.

**Table 3:** Comparison of laser surface treatments for enhancing zirconia bonding Er laser: erbium laser; Nd laser: neodymium laser; CO_2_ laser: carbon dioxide laser

Study	Laser Type	Effect on Surface	Impact on Bond Strength	Advantages	Disadvantages
Bitencourt et al. [47] Arami et al. [49] Jauregui-Ulloa & Marocho [50]	CO_2_ laser	Creates micro-retentive features through thermomechanical ablation	Enhances micromechanical retention and improves shear bond strength	High precision; maintains zirconia integrity	Needs careful energy management to avoid thermal damage
Bitencourt et al. [47] Du & Niu [48] Arami et al. [49]	Nd laser	Surface roughness via micro-explosions, localized melting	Increases surface roughness	Effective at moderate energy; creates a rough surface	High-energy settings cause microcracks and defects and reduce long-term stability
Bitencourt et al. [47] Du & Niu [48] Arami et al. [49]	Er laser	Micro-explosions create surface roughness	Less effective than CO_2_ and Nd lasers for improving bond strength; fails to create significant retention points	Less risk of surface damage at lower power levels	Insufficient roughness for strong mechanical interlocking
Jauregui-Ulloa & Marocho [50] Yilmaz-Savas et al. [52]	Femtosecond laser	Ultrafast laser pulses, precise surface modifications	Highly effective, creates structured roughness	Minimal thermal damage, high precision	Expensive and complex; not widely available
Sahoo et al. [51]	Ultrashort pulse laser	Short, precise pulses create micro-retentive surface features	Enhances bond strength without damaging zirconia structure	No thermal damage, improved wettability and roughness	Costly and requires precise equipment

SIE: a superior surface treatment for zirconia

SIE, as investigated by Aboushelib et al. [55], is a cutting-edge surface treatment designed to improve the bond strength between zirconia and resin cements. SIE works by manipulating the surface structure of zirconia, creating nano-retentive intergrain porosities that enhance mechanical retention and bonding strength. The process involves applying a glass-based conditioning agent to the zirconia surface, which is then heated above the glass transition temperature. This allows the glass to infiltrate the zirconia surface, facilitating grain boundary diffusion, sliding, and rearrangement. Ultimately, it creates the porosities necessary for adhesion. Afterward, the glass is removed using an acidic bath, leaving behind a highly retentive surface [55].

When comparing the bond strength of SIE-treated zirconia to untreated and airborne-particle abraded zirconia, SIE demonstrated superior microtensile bond strength (MTBS). It maintained values as high as 51.9 MPa, even after extensive artificial aging via thermocycling and water storage [55]. In contrast, bond strength in the APA group deteriorated over time, and untreated zirconia exhibited poor initial and post-aging bond strength. Nanoleakage tests further confirmed SIE's superior performance, as treated zirconia provided a better seal at the zirconia-resin interface, preventing silver nitrate infiltration, an important indicator of bonding stability [55].

Casucci et al. [56] further investigated SIE as a surface treatment for zirconia ceramics. After applying a glass-containing agent to the zirconia surface and heating it to approximately 750°C, the researchers found that SIE infiltrated the grain boundaries. Once the glass was removed with hydrofluoric acid, a micro-retentive, roughened surface remained, which is ideal for bonding with resin cements. Casuccia's study found that SIE-treated zirconia samples exhibited significantly higher MTBS compared to traditional treatments such as sandblasting, a result attributed to the nano-retentive porosities created during the process [56]. SEM analysis confirmed that SIE produced a rougher surface with more retentive features than sandblasting and therefore enhanced mechanical retention. Notably, adhesive failures were more common in untreated and sandblasted groups, whereas SIE-treated groups exhibited mixed failures, indicating stronger bonds between zirconia and resin cement [56].

Jiang et al. [57] conducted a study comparing SIE with conventional treatments such as APA. Their findings revealed that SIE-treated zirconia exhibited significantly higher surface roughness and bond strength than untreated or APA-treated groups. The roughness values (Ra) for SIE-treated zirconia reached 12.42 μm, indicating better mechanical interlocking with resin cements. Even after 10,000 thermocycling cycles, SIE-treated zirconia maintained bond strength, while untreated zirconia showed a marked decline in performance, emphasizing SIE's durability and stability [57]. SEM analysis further supported this by showing micro-retentive spaces between zirconia grains post-SIE treatment, which contributed to stronger adhesive bonding with resin cements.

Lv et al. [58] reinforced the advantages of SIE over traditional treatments. The study compared SIE with APA, highlighting that SIE significantly improved both surface roughness and SBS. The infiltration process created nano-porous structures that enhanced micromechanical retention and offered better long-term bond durability than APA, which can cause surface damage and microcracks. SIE also demonstrated superior resistance to thermal cycling degradation, a critical factor for maintaining bond integrity in the oral environment [58].

Casucci et al. [59] expanded on these findings by comparing the effects of SIE, APA, and other etching methods on the surface morphology of various zirconia ceramics. SIE-treated surfaces exhibited the highest surface roughness and the best micromechanical retention. Atomic force microscopy (AFM) and SEM images illustrated the creation of intergrain porosities after SIE treatment, significantly improving MTBS [59].

Jiang et al. [60] also examined SIE's impact on surface roughness and bond strength in zirconia ceramics. Their study confirmed that SIE-treated zirconia exhibited higher surface roughness than untreated controls, providing a durable and stable bond. In contrast to more aggressive treatments such as APA, which can cause microcracks, SIE preserves zirconia's long-term mechanical stability and avoids such damage [60].

Across all studies, SIE consistently outperformed traditional surface treatment methods, such as APA, by creating a more durable and retentive surface for bonding with resin cements. SIE's ability to form nano-retentive intergrain porosities, combined with its resistance to nanoleakage and degradation after thermocycling, makes it a superior technique for improving bond strength and long-term stability in zirconia restorations. Unlike APA, which can induce surface damage and microcracks, SIE enhances surface roughness without compromising structural integrity. This ability to create a stable interface that resists nanoleakage and thermal cycling makes SIE a highly effective surface treatment for zirconia, especially in clinical applications where long-term durability is critical.

Cements used for zirconia: a key component in bonding success

The clinical success and longevity of zirconia restorations, for both full and partial prostheses, depend heavily on the cementation protocol used. A strong cement-zirconia bond not only improves retention but also ensures better marginal adaptation and resistance to fracture. In the cementation of any prosthesis, two critical interfaces exist: the dentin-cement interface and the cement-prosthesis interface. Testing the SBS between these two interfaces is an essential component of bonding efficacy evaluations. Studies by Piwowarczyk et al. [61] compared conventional cements, such as zinc phosphate and glass ionomer, with resin cements, including those containing 10-MDP. The results showed that both resin and MDP-containing cements exhibited superior bond strengths compared to conventional cements, particularly after aging and thermocycling.

In addition, Lüthy et al. [62] found that glass ionomer and BisGMA cement had lower bond strengths compared to resin cement. While in vitro studies clearly favor resin cements due to their superior adhesion, clinical studies present a more complex picture. Interestingly, the type of cement did not significantly influence the clinical success and survival of full-coverage zirconia restorations on both teeth and implants [63-67]. This discrepancy between in vitro and in vivo findings highlights the complexity of real-world applications and the need for tailored clinical solutions. Resin cements are typically classified into self-adhesive cements, cements containing 10-MDP, and cements containing BisGMA. Of these, BisGMA cements have been consistently noted to have lower adhesion values [68,69] despite their high initial bond strengths. However, as Wegner and Kern [70] observed, the bond strength of BisGMA cements tends to decline over time. In contrast, 10-MDP-containing cements have been found to produce a synergistic effect, especially when used with self-adhesive cements [71-73]. Non-MDP self-adhesive cements, conversely, benefited significantly from the application of a zirconia primer; whereas, MDP-containing cements did not exhibit a similar increase in bond strength after primer application [72].

A study by Le et al. [74] explored the impact of cross-system primer and cement combinations on the bond strength between zirconia and dentin. The researchers compared RelyX Ultimate (3M, Saint Paul, MN, USA) with Panavia V5 (Kuraray Noritake, Tokyo, Japan) and found that using Panavia's specialized primers with Panavia cement resulted in the highest bond strength. Interestingly, combining Panavia primers with RelyX cement also produced strong bonds, although not as high as when paired with Panavia's own cement. In contrast, cross-system combinations involving Panavia primers and Scotchbond Universal Adhesive from RelyX yielded significantly lower bond strengths. These findings emphasize the critical importance of adhering to manufacturer-recommended pairings for optimal bonding outcomes. While some cross-system combinations can still yield effective results, the specialized formulations of primers, such as Panavia's, designed specifically for zirconia, play a key role in fostering durable adhesion. As multipurpose adhesives such as Scotchbond may not perform as effectively when used outside their intended systems, it is risky to deviate from the recommended product combinations.

Role of Surface Treatments in Cement Selection

The choice of cement is intricately related to the surface treatments applied to zirconia. As discussed earlier, chemical surface modifications such as silica coating, tribochemical silica coating, and Zircos-E multi-acid etching all play pivotal roles in enhancing micromechanical retention. These treatments significantly roughen the zirconia surface and create a more reactive topography that aids in bonding to resin cements. For instance, the silica coating chemically interacts with silane coupling agents to provide an additional layer of chemical bonding to improve retention [22]. Similarly, Zircos-E enhances micromechanical retention by creating a microporous surface, which increases surface area and promotes stronger resin cement interactions [33-35]. When using MDP-containing cements, especially in combination with surface treatments such as APA, the interaction between the phosphate group in 10-MDP and the zirconia's oxide layer becomes critical. This is because 10-MDP cements form strong P-O-Zr bonds, enhancing bond strength and providing a stable, long-lasting interface, particularly in resisting hydrolytic degradation [39,42]. These bonds, which are more resistant to moisture, ensure that the bond between zirconia and resin remains durable over time. Furthermore, these chemical interactions are complemented by mechanical surface roughening, such as the increase in surface energy created by APA, which promotes better prosthesis retention and long-term stability [36-39].

Optimizing Cement-Primer Combinations

The study by Le et al. [74] emphasized the importance of using the right primer-cement combination. While Panavia's system worked well both within its own system and with other cements such as RelyX, the reverse did not hold true. This demonstrates that some primer-cement combinations are optimized for specific chemical interactions, particularly for surface treatments such as silica coating [22] or Zircos-E [47]. For example, Panavia's primers, which are formulated specifically for zirconia, excel in these combinations due to their strong phosphate-based chemical bonding mechanism [36-39]. Conversely, universal adhesives such as Scotchbond, which are designed for multiple materials, may not provide the same level of specialized bonding to zirconia when combined with other systems [72].

This distinction becomes especially important in clinical practice, where selecting compatible products is crucial for long-term success. Cross-system combinations, although sometimes effective, often result in reduced bond strengths if manufacturers' recommendations are not followed [72-74]. For example, studies have shown that the use of primers containing 10-MDP with self-adhesive cements produces a synergistic effect that improves bond durability [71-73]. However, non-MDP-containing cements may still require the application of a zirconia primer to achieve adequate bond strength [71].

These findings underscore the need for clinicians to adhere closely to manufacturer guidelines when selecting and applying bonding systems. Failing to do so could compromise the long-term durability of zirconia restorations, especially in cases where aging conditions such as thermocycling play a role in bond degradation [39,42].

Long-term clinical considerations

The right cement selection plays a pivotal role in the long-term success of zirconia restorations. Resin cements, particularly those containing 10-MDP, consistently demonstrate superior bond strength and retention compared to conventional cements such as glass ionomer and zinc phosphate [61,63]. However, the clinical implications of these findings are not always straightforward. While in vitro studies strongly favor resin cements, clinical trials suggest that the type of cement does not always significantly affect the survival rate of zirconia restorations [64-67]. The performance of resin cements is heavily dependent on several other factors, such as surface treatment, primer-cement compatibility, and aging conditions such as thermocycling [39,72]. Cross-system combinations of primers and cements, while sometimes effective, often lead to reduced bond strengths if manufacturer guidelines are not strictly adhered to [74]. This emphasizes the importance of selecting the right products and following the manufacturers' instructions to ensure strong and durable bonding outcomes. By integrating the choice of cement with appropriate surface treatments, especially those optimized for specific chemical interactions, clinicians can achieve better long-term results in zirconia restorations [22,36]. Further research is essential to continue refining cementation protocols and developing solutions that perform consistently across various clinical conditions.

New and upcoming research

He and Swain [75] introduced polymer-infiltrated ceramic networks (PICNs) in dentistry over a decade ago. PICNs are porous ceramic/resin composites created by infiltrating resin monomers into a ceramic structure, which are then polymerized. Although designed to combine the strengths of ceramics and composites, traditional PICNs have been limited by their low flexural strength. To overcome this limitation, zirconia-based PICNs have been explored due to their enhanced mechanical properties, making them promising candidates for restorative materials. Ikemoto et al. [76] fabricated an experimental zirconia-based PICN that demonstrated superior mechanical properties, such as improved flexural strength (346 MPa) and hardness, compared to traditional silicate-based PICNs. The material's bonding properties, water sorption, and solubility were also comparable to those of resin composites [76]. However, Li and Sun [77] reported a lower flexural strength of 211.8 MPa for a similar zirconia-based PICN. The discrepancy between these studies is likely due to differences in fabrication techniques: Ikemoto et al. [76] used slip casting, while Li and Sun [77] employed cold isostatic pressing [77].

Despite the promising mechanical properties, zirconia-based PICNs have limitations, such as their white color, which necessitates the use of coloring agents to mimic natural tooth shades. Additionally, further research is needed to assess the biocompatibility, machinability, and optical properties of these materials before clinical adoption. Current research lacks long-term clinical studies, making the performance of zirconia-based PICNs in the oral environment still uncertain. Future clinical trials are essential to establish their applicability in dental restorations.

Another emerging field is additive manufacturing (AM) in zirconia. A systematic review by Alghauli et al. [78] compared AM zirconia with conventionally milled zirconia, highlighting no mechanical or biological complications with AM 3Y-TZP crowns in short-term clinical trials [78]. Additional in vitro studies reviewed by the group assessed various properties of 3D-printed zirconia, including mechanical performance, trueness, accuracy, precision, biological response, and aesthetics [79]. Despite limited mechanical performance in thinner samples, 3D-printed zirconia shows significant potential. However, as with zirconia-based PICNs, long-term clinical studies are required to fully evaluate their clinical potential and optimize manufacturing techniques. Both zirconia-based PICNs and AM zirconia represent promising advancements in dental restorative materials. While early studies are encouraging, further research is necessary to confirm their long-term performance and clinical suitability, ensuring they can be effectively integrated into existing dental protocols to enhance patient outcomes.

## Conclusions

The current dental literature presents various protocols for the cementation of zirconia prostheses with differences in surface treatments, primers, and cements. However, comparing the results of different studies is challenging due to the predominance of in vitro studies employing different testing parameters. Consequently, a few conclusions can be drawn. First, a combination of mechanical and chemical treatment is essential for good adhesion. APA with 50 μm alumina (Al_2_O_3_) particles is the most commonly used method. In addition, a zirconia primer containing 10-MDP is essential, even though the choice of cement is not critical for bonding success. If resin cement is used, light curing should be done for a sufficient duration (40 seconds/surface). Rubber dam isolation is also recommended for zirconia bonding. Finally, further long-term clinical research is required to establish uniform guidelines for bonding to full and partial coverage zirconia restorations.
